# A Giant Posterior Maxillary Intra-Sinusal Complex Odontoma: A Clinical Case

**DOI:** 10.7759/cureus.42546

**Published:** 2023-07-27

**Authors:** Mohamed Kadri, Aude Grand, Marine Mondoloni, Paul Walter, Soufiane Boussouni, Juliette Rochefort

**Affiliations:** 1 Department of Odontology, Health Faculty, Université Paris Cité, Assistance Publique-Hôpitaux de Paris (AP-HP) Pitié-Salpêtrière Hospital, Paris, FRA

**Keywords:** oroantral communication, complex odontoma, odontogenic tumors, impacted teeth, computed tomography

## Abstract

Odontomas are the most common odontogenic neoplasms. They are generally small and asymptomatic. This article presents an unusual case of a giant maxillary complex odontoma, which obscured a part of the maxillary antrum and impacted a tooth. This was discovered during an episode of maxillofacial cellulitis. In this case, surgical excision of the lesion was performed under general anesthesia, and the closure was performed with a fat pad pedicled flap. A brief review of the literature was performed to analyze the characteristics of this clinical entity and their implication in the treatment.

## Introduction

An odontoma is currently considered as a hamartoma because it is highly differentiated. It is a mixed odontogenic benign tumor because it is both epithelial and mesenchymal in origin [1]. According to the 2017 World Health Organization classification, there are two types of odontomas: complex odontomas, which consist of an anarchic assembly of mineralized tissue (enamel, dentin, and cementum) and pulp, and compound odontomas, which present as more differentiated tissue in the form of multiple small teeth [1]. Hybrid forms such as cystic odontomas have also been described. The average age of diagnosis of complex odontomas is approximately 26 years, with a male predominance [2]. Most odontomas described in the literature are between 1 and 2 cm in diameter [3]. Beyond this size, they are called giant odontomas [4].

The aim of this report is to describe a case of giant maxillary intrasinusal complex odontoma revealed by cellulitis and to describe the specifics of treatment resulting from this localization. We have also analyzed similar cases that have been reported in the literature and discussed the importance of imaging.

## Case presentation

A 27-year-old patient with no allergy presented with right posterior maxillary severe pain (visual analogue scale [VAS] score = 80/100). He had no medical or surgical history. No radiological examination was prescribed at the time.

**Figure 1:**
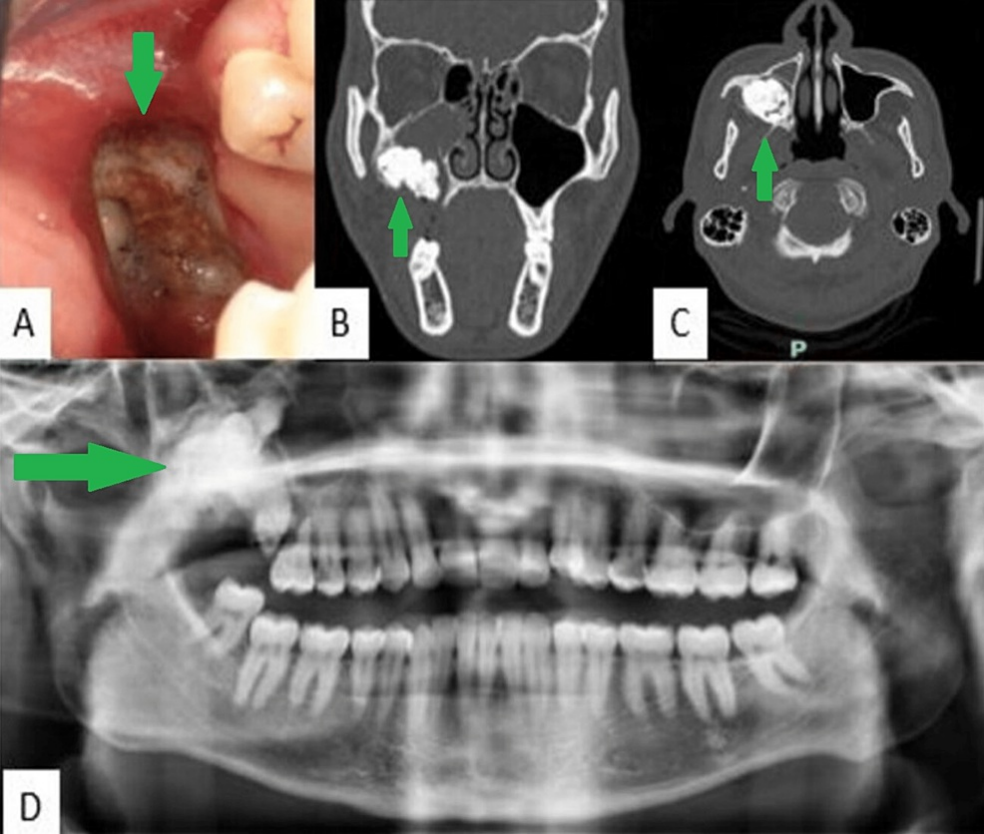
(A) Clinical appearance after biopsy. CT scan in the (B) coronal and (C) axial sections of the hyperdense mass, which found a 33 x 23 x 21 mm right intra-sinusal encysted bone density mass with inclusion of the probable teeth 17 and 18. (D) Panoramic radiograph on the day of the first consultation showing an inhomogeneous radiopaque image surrounded by a radiolucent halo at the level of the tuberosity and the right maxillary sinus.

On the day of the consultation, facial examination revealed a swelling in the right upper jaw, which was firm, non-indurated, and without eyelid edema. Endobuccal examination revealed a fluctuating swelling from distal to tooth 16 associated with exposure of hard tissue of inhomogeneous appearance extending 2 cm up to the right maxillary tuberosity. The panoramic radiograph revealed a heterogeneous radiopaque image of about 4 cm in its long axis, well delimited by a radiolucent halo (Figure 1). The diagnosis of cervico-facial cellulitis was retained, and an antibiotic therapy based on amoxicillin 1 g (two tablets per day for six days) and metronidazole 500 mg (three tablets per day for six days) was prescribed for six days as well as level II analgesics (paracetamol codeine 500/30 mg, two tablets every six hours in case of pain) and a local antiseptic (chlorhexidine 0.12%) in the form of mouthwash. A computed tomography (CT) scan was also ordered. At the follow-up visit, seven days later, the CT evidenced a hyper-dense mass, which found a 33 x 23 x 21 mm right intra-sinusal encysted bone density mass with inclusion of the probable teeth 17 and 18 (Figure 1).

The diagnostic hypothesis that was considered was a giant and complex odontoma. The differential diagnosis for this lesion included calcifying odontogenic cyst, calcifying odontogenic tumor, ameloblastic fibroma, fibro‑odontoma, and fibro‑osseous lesion. Given these different diagnostic hypotheses, a biopsy was performed. The pathological examination concluded that the patient had a complex odontoma without any sign of malignancy.

**Figure 2:**
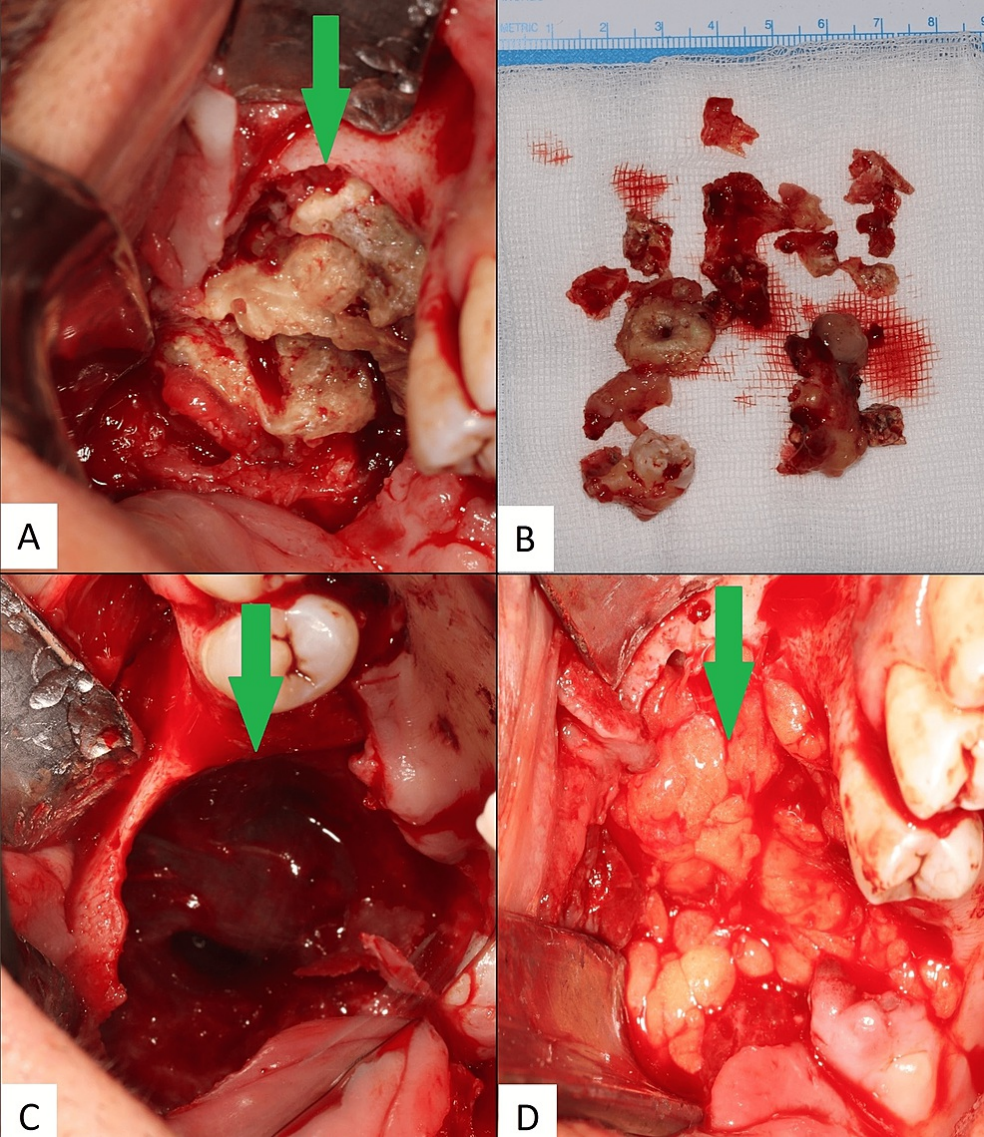
A conservative exeresis of the lesion was made with intra-oral access. (A) Identification of the lesion. (B) Fragmented odontoma. (C) Overview of the buccal-sinusal communication. (D) Pedicled buccal fat pad flap.

The complete exeresis was performed for a second time under general anesthesia. Given the size of the mass, the closure was performed with the buccal-sinusal communication by a pedicled buccal fat pad flap (Figure 2).

**Figure 3:**
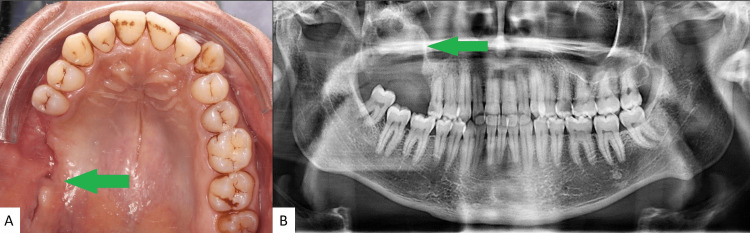
(A) Clinical appearance six weeks after surgery showing mucosal healing and bucco-sinusal closure. (B) Dental panoramic six weeks after surgery showing the beginning of bone healing.

The one-month follow-up showed mucosal healing and the absence of oral-sinusal communication. On panoramic radiography, we can see the beginning of bone healing with the presence of a radiopaque border (Figure 3). Five-month follow-up showed complete healing.

## Discussion

Most odontomas are asymptomatic and may present as a slowly evolving painless expanding process. They can be detected either fortuitously during routine radiological examinations, or either in the context of delayed dental evolution, or in case of an infectious episode. Delays in diagnosis of these conditions can lead to abnormalities in tooth eruption or even structural abnormalities of adjacent teeth. For this reason, a radiological check-up should always be performed in case of an eruption disorder and before each tooth extraction [1].

In this case, the odontoma was present in the maxillary tuberosity which is a very rare location (<2%) [3]. Indeed, odontomas are most often located in the anterior maxilla and in the parasymphyseal region of the mandible [3]. Intra-sinus and tuberosal localizations are very rare. The majority of cases of giant complex odontomas reported in the literature are located in the mandible and were asymptomatic complex odontomas [4-6].

Four relevant articles on the subject were reviewed, which contained four case reports of giant maxillary complex odontomas [7-10]. We have reported clinical and radiological characteristics of these cases in Table 1. The data collected in the articles [7-10] showed that the average age of the patients reported in the literature was 22 years and that the male-to-female ratio was 3:1. Only one patient out of the four presented with pain, and this same patient had an oral exposure of the lesion [9]. In three out of four clinical cases, the location of the lesion was the maxillary sinus [8-10]; only one case reported lesion in the central maxillary region [7]. Three of the four cases had extra-oral swelling, but all cases were associated with dental eruption anomalies. These delays in tooth eruption associated with extra- and intra-oral manifestations have led patients to consult dentists to refer them to specialized services. The use of three-dimensional (3D) radiological examination was essential for the management of the patients, which consisted in the removal of the lesion.

**Table 1:** Description of giant complex odontomas of the maxillary reported in the literature. CBCT, cone beam computed tomography

Study, year	Gender Age	Diameter	Pain	Oral exposition	Location	Radiologic description	Clinical aspect
Saurabh et al., 2015 [9]	M, 25	45 mm	Painful	+	Maxillary sinus	Panoramic: irregular, radiopaque lesion; CBCT: hyperdense mass in the left maxillary sinus	Swelling over the left side of the cheek region
Carvalho Visioli et al., 2015 [8]	M, 21	50 mm	Painless	-	maxillary sinus	Panoramic: solid radiopaque mass involving left alveolar ridge, maxillary sinus, and orbit	Absence of the maxillary left second and third molars; swelling of the alveolar process
Utumi et al., 2011 [7]	F, 10	40 mm	Painless		Central maxillary region	Panoramic: radiopaque image in the area of the permanent maxillary right central incisor; CBCT: scan shows an irregular circumscribed lesion in the hard palate	Absence of the maxillary right central incisor and enlargement of the anterior region of the hard palate and maxilla
Isler et al., 2009 [10]	M, 33	45 mm	Painless		Maxillary sinus	Panoramic: giant radiopaque mass caused resorption at the root of the first molar; CBCT: 4.5 x 4 x 3 cm hyperdense mass, extending from the alveolar ridge to the middle third of the maxillary sinus	Intra-oral inspection revealed a significant expansion along both the buccal and palatine aspects. The mass had erupted into the oral cavity and was covered by attached gingiva.

The clinical presentation of this lesion type is highly variable, depending on the patient. Exo-buccal swelling may or may not be present. During an endobuccal examination, delayed tooth eruption and a buccal exposure of the lesion with an inhomogeneous appearance may be observed.

Dental panoramic imaging reveals an inhomogeneous radio-opaque mass. Further analysis must be conducted using CT scans to precisely determine the appearance, extent, and relationship to surrounding structures and to establish a definitive diagnosis and rule out differential diagnoses such as calcifying odontogenic cyst, calcifying odontogenic tumor, ameloblastic fibroma, fibro-odontoma, and fibro-osseous lesion. Furthermore, a biopsy and an anatomopathological examination must be performed. Prior to the removal of a maxillary giant odontoma, a comprehensive treatment plan with oral rehabilitation solutions is necessary.

Some authors have shown that an odontoma, giant or not, can interfere with eruption of the adjacent teeth, causing impaction [11]. This requires dental restoration or filling of a large edentulous tooth depending on the size of the giant odontoma, the location of the lesion, and the number of teeth affected. These formations, although benign, can be very destructive and mutilating. They require a maxillofacial prosthesis or a consequent bone reconstruction, especially in the maxillary posterior sectors.

The etiology of this disease is still unknown, but many factors have been considered to play an important role in its pathogenesis, such as local trauma, infection, genetics, or a family history of conditions such as Gardner syndrome and Hermann syndrome [4,7].

The 3D-CT examination is important for the correct diagnosis of the expansion and involvement of the tumor. The exact position of all involved structures (maxillary sinus, maxillary molars, alveolar ridge, and hard palate) was verified by 3D-CT, allowing for the best planning of the surgical intervention [11]. To our knowledge, there are no recommendations for the management of these structures.

## Conclusions

Through this article, we understand the importance of systematic complementary examinations (panoramic radiography, CT scan) to avoid diagnostic errancy particularly when faced with eruption anomalies. Although these are benign tumors, their removal can be very mutilating and should be combined with other surgical techniques such as closure of the buccal-sinusal communication with a fat pad pedicled flap in this case. When the damage to the adjacent teeth and surrounding bone is severe, complex oral rehabilitation is required; hence, multidisciplinary therapeutic strategies are important.
